# New ovulate cupule further informs the relationships among early seed plants and their adaptation to wind pollination

**DOI:** 10.1098/rspb.2024.2940

**Published:** 2025-03-26

**Authors:** Bing-Xin Li, Pu Huang, Le Liu, Jia-Shu Wang, Karl Niklas, De-Ming Wang, Jin-Zhuang Xue

**Affiliations:** ^1^ The Key Laboratory of Orogenic Belts and Crustal Evolution, School of Earth and Space Sciences, Peking University, Beijing 100871, People’s Republic of China; ^2^ State Key Laboratory of Palaeobiology and Stratigraphy, Nanjing Institute of Geology and Palaeontology, Chinese Academy of Sciences, Nanjing 210008, People’s Republic of China; ^3^ School of Geoscience and Surveying Engineering, China University of Mining and Technology (Beijing), Beijing 100083, People’s Republic of China; ^4^ Laboratory of Geo-specimens Study and Testing, Geological Museum of China, Beijing, People’s Republic of China; ^5^ School of Integrative Plant Science, Cornell University, Ithaca, NY 14853, USA

**Keywords:** seed plants, cupule, wind pollination, anemophily, computational fluid dynamics simulation, Devonian

## Abstract

The earliest seed plants occurred in the Late Devonian (Famennian). However, why and how they rapidly diversified remain long-standing mysteries. We investigated the early evolution of seed plants based on a new ovule and evaluated wind pollination performance of the earliest cupulate ovules by using computational fluid dynamics simulations. *Zaijunia biloba* gen. et sp. nov. is described from the Upper Devonian (Famennian) of South China and shows canonical hydrasperman-type ovules with lobed integuments and a complex nucellar apex. *Zaijunia* bears ovules in pairs, each ovule within a lateral bilobed cupule. We propose that duplication of the bi-ovulate fertile unit of *Zaijunia* could produce more complex derivative cupulate ovules, as an evolutionary pathway leading to the early diversification of seeds. We performed computational fluid dynamics simulations of *Zaijunia* and two other early seed plants (i.e. *Pseudosporogonites* and *Elkinsia*), demonstrating that their fertile units improve airborne (pre)pollen capture efficiency. This study sheds additional light on the evolution of cupulate ovules and their potential adaptations to anemophily, as one of the factors driving the earliest radiation of seed plants.

## 1. Introduction

The emergence of the seed habit has been considered one of the most important events in land plant evolution [1–4]. Seed plants first appeared in the Late Devonian (Famennian) and show an initial rapid radiation, as evidenced by more than 30 fossil genera mainly described from North America, Europe and South China [5,6]. The effect of early seed plants on the evolution of Earth’s terrestrial ecosystems, via their ability to adapt to a broader spectrum of habitats, including arid and semi-arid habitats, compared with free-sporing plants, has been repeatedly emphasized [7,8].

The earliest ovules/seeds consist of a single functional megaspore contained within an indehiscent megasporangium (nucellus) surrounded by an integument of various morphologies, with or without a surrounding cupule [2,6,9–13]. It has been suggested that early seed plants were wind pollinated, before insects diversified and began to feed on ovule secretions or (pre)pollen organs, as shown in the fossil record [14,15]. However, complex interactions between potential pollinators and seed plants have been demonstrated in Early Permian fossils [16] and might have evolved much earlier [15]. Consequently, the assumption that all early seed plants relied on wind pollination is subject to well informed debate. In the Mesozoic, the diversification of flowering plants has been linked with the innovations involving their coevolution with pollinators as well as herbivores [17,18]. As occurred much later in the evolution of early flowering plants, the first seed plants also appear to have experienced a remarkably rapid rise and diversification in the morphology of the integument and cupule in the Late Devonian. However, why and how they rapidly diversified remain mysterious.

In this article, we describe a new hydrasperman seed plant, *Zaijunia biloba* gen. et sp. nov., from the Upper Devonian (Famennian) of China, based on a rich collection of stems, fronds and cupulate ovules. The morphological comparison between this new plant and the well studied seed plant *Elkinsia* [6,9], as well as its presumptive relatives such as *Archaeosperma* [19] and *Pseudosporogonites* [11], enables us to recognize the most primitive architecture of cupulate ovules. In addition, computational fluid dynamics (CFD) allows us to explore if these Late Devonian seed plants had the capacity for wind pollination and to what degree, which may have contributed to their early rapid diversification.

## 2. Material and methods

### (a) Specimens

**Figure 1.**
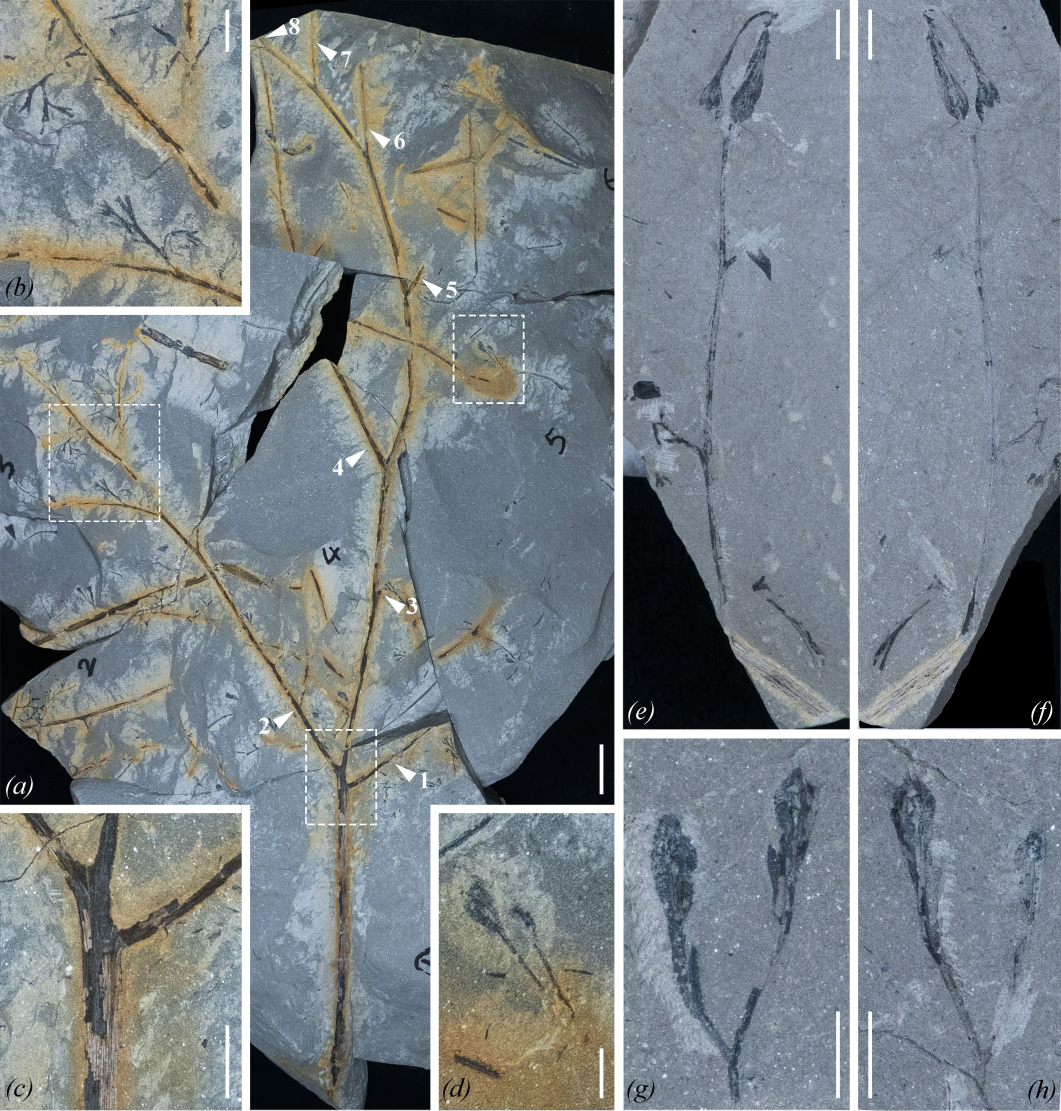
Morphology of *Zaijunia biloba* gen. et sp. nov. (*a*) Primary rachis, bearing at least eight helically arranged secondary pinnae (SPs) (white arrowheads with numerals 1−8), which in turn bear tertiary pinnae. Note the third SP (numeral 3) extends towards the readers and is broken, with only its base preserved. PKUB20184. (*b*) Enlargement of a part of (*a*) (left rectangle), showing secondary and tertiary pinnae and pinnules. (*c*) Enlargement of a part of (*a*) (lower rectangle), showing attachment of the two most proximal SPs. Longitudinal striations distributed along primary rachis and SPs. (*d*) Enlargement of a part of (*a*) (upper right rectangle), showing two poorly preserved, detached ovules. (*e*,*f*) Part and counterpart of a fertile pinna. Ultimate rachis shows a terminal fertile unit composed of two cupulate ovules, and proximally, two laterals that are shown as broken bases. Penultimate rachis shows longitudinal striations (bottom of the photographs). PKUB20123A, B. (*g*,*h*) Part and counterpart of a fertile unit that shows a basal fork, producing two segments that are unequal in length; each segment is terminated by a cupulate ovule. PKUB20121A, B. Scale bars, 20 mm (*a*), 10 mm (*b,c*) and 5 mm (*d–h*).

**Figure 2.**
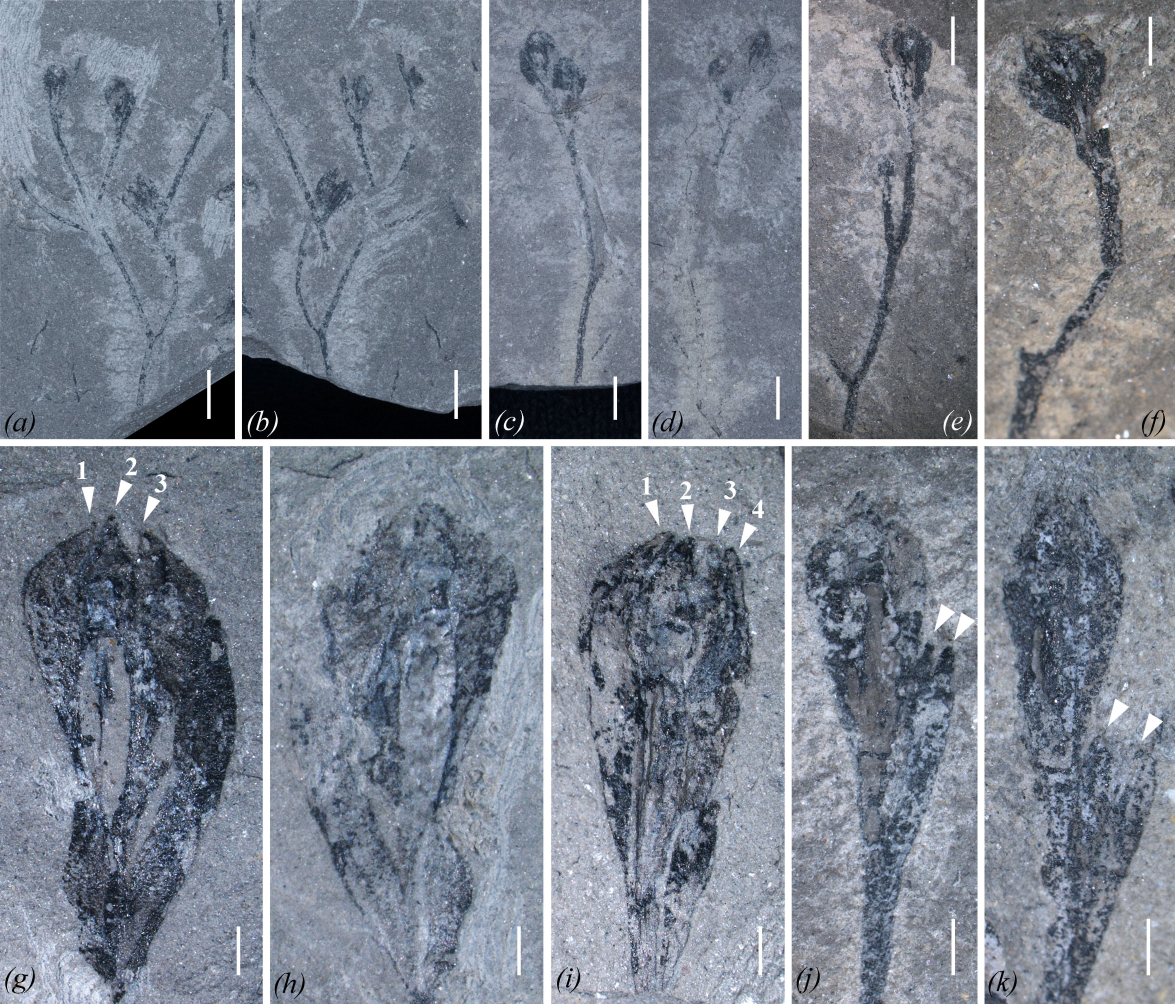
Fertile axes and ovules of *Zaijunia biloba* gen. et sp. nov. (*a,b*) Part and counterpart of a cupule-bearing system, which divides once and bears two fertile units; each unit is composed of a pair of cupulate ovules. PKUB20128A, B. (*c*,*d*) Part and counterpart of a fertile unit comprising a pair of cupulate ovules. PKUB20169A, B. (*e*) Dichotomous cupule-bearing system with a single distal ovule, while others are broken. PKUB20210. (*f*) Terminal ovule. The other ovule of a pair is broken. PKUB20233. (*g*,*h*) Part and counterpart of an ovule with three integumentary lobes (arrowheads with numerals 1−3) visible and a cupule. Note that one side of the ovule is distorted and that in (*g*) there is a lagenate nucellus trace. PKUB20100A, B. (*i*) Ovule with four integumentary lobes visible (arrowheads with numerals 1−4) and a cupule at right side. PKUB20114. (*j*,*k*) Ovules, each subtended by a bilobed cupule. Arrowheads point to the two free tips of the cupules. PKUB20153, PKUB20141. Scale bars, 5 mm (*a,b*), 2 mm (*c,d*) and 1 mm (*e–k*).

**Figure 3.**
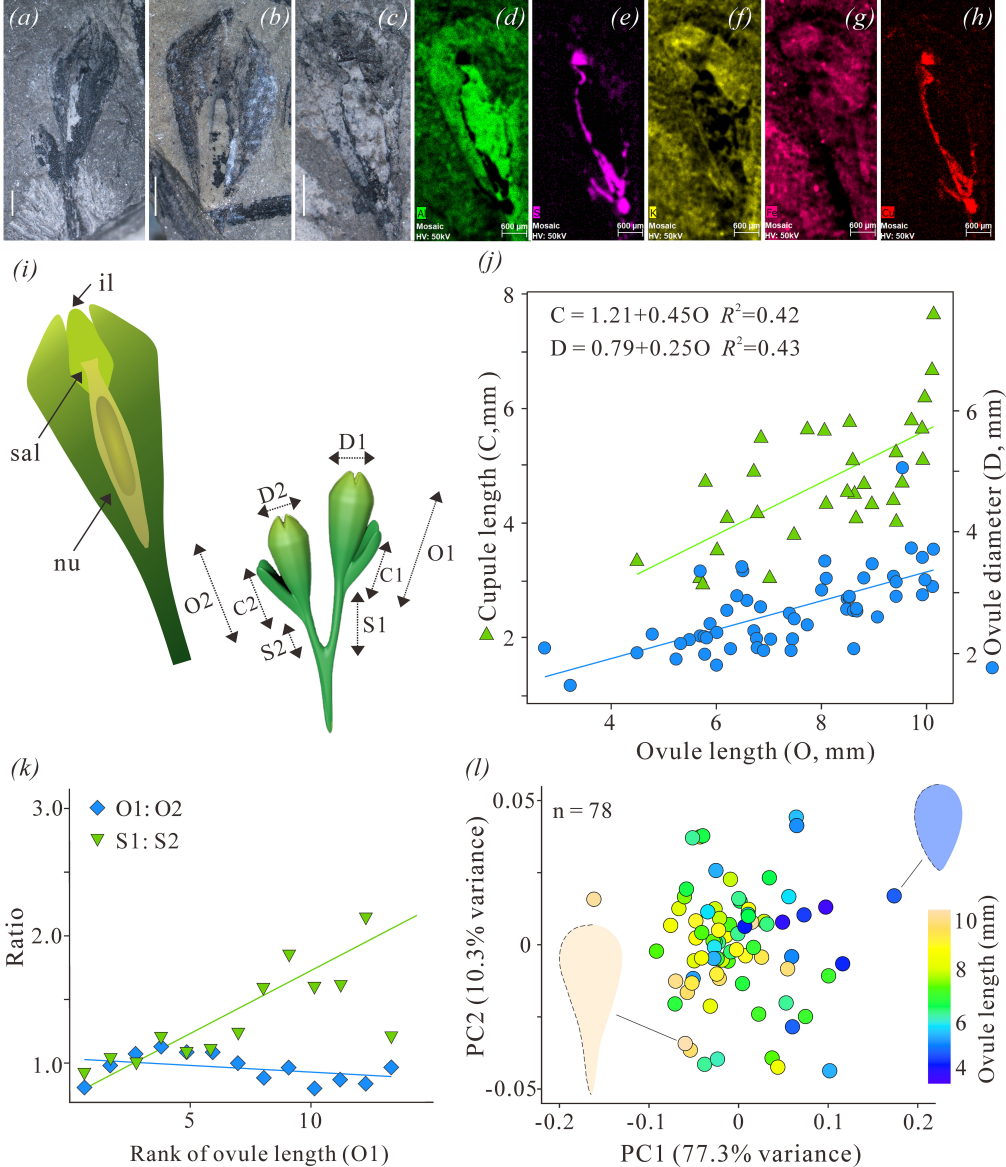
Ovules of *Zaijunia biloba* gen. et sp. nov. and their measurements. (*a*) Holotype. Ovule with integumentary lobes and a bilobed cupule at left side. PKUB20191. (*b*) Ovule with a lobed integument and a nucellus trace, showing salpinx and pollen chamber. PKUB20190. (*c*) Ovule with a nucellus trace, showing the shape of nucellus. PKUB20170. (*d***–***h*) Elemental mapping of aluminium (Al), sulfur (S), potassium (K), iron (Fe) and copper (Cu) of the ovule in (*c*), by using micro X-ray fluorescence spectroscopy. (*i*) Models and descriptors of a fertile unit (right) and an individual ovule (left). il, integument lobe; sal, salpinx; nu, nucellus; C1 and C2, length of the two cupules of a fertile unit; D1 and D2, diameter of the two ovules of a fertile unit; O1 and O2, length of the two ovules; S1 and S2, length of the two stalks of the ovules. (*j*) Cross-plot between ovule length and ovule diameter, and between ovule length and cupule length. O = both O1 and O2, D = both D1 and D2, and C = both C1 and C2 in (*i*). (*k*) The changes of O1 : O2 ratio and S1 : S2 ratio of each pair of ovules, according to the rank of O1. (*l*) Principal component analysis results of the outline of ovules in different growth stages. Ovule length (mm): different lengths of ovules shown by different colours. The outlines of the smallest ovule and one of the largest ovules are shown, i.e. PKUB20128 (upper right) and PKUB20141 (lower left), respectively. Scale bars: 1 mm (*a–c*); 600 µm (*e–g*).

About 250 specimens were collected in 2013 from the Leigutai Member of the Wutong Formation at an abandoned quarry near Guangyao village, Xiaopu town, Changxing county, Zhejiang province, China (electronic supplementary material, figures S1 and S2, Guangyao section; figures 1–3). All specimens are from a single fallen rock block of grey siltstone, belonging to the lower part of the Leigutai Member. Based on spore assemblages, Ouyang [20] suggested that the uppermost part of the Wutong Formation is Tournaisian, whereas lower beds, including the Leigutai Member, are of late to latest Famennian, a scheme followed by most subsequent studies. We consider that the Guangyao section can be correlated with the nearby Fanwan section (electronic supplementary material, figures S1 and S2), where very rich plant assemblages, including *Archaeopteris*, *Sublepidodendron* and *Eviostachya*, have been described [12,21].

Steel needles were used to expose fronds, fertile axes, cupules and ovules. Large specimens were photographed with a digital camera, and fine structures were photographed under a stereomicroscope connected with a digital camera system.

All specimens are housed at the Geological Museum, School of Earth and Space Sciences, Peking University, Beijing, China, and bear collection numbers PKUB20100 to PKUB20258.

### (b) Micro-X-ray fluorescence spectroscopy analysis

The elemental maps of two ovules were obtained by using an M4 Tornado micro-X-ray fluorescence (µ-XRF) spectroscope (Bruker, Germany) (PKUB20170: figure *3c–h*, and electronic supplementary material, figure S8; PKUB20155: electronic supplementary material, figure S9). The source of the M4 Tornado consists of a 30 W Rh anode metal–ceramic X-ray tube with maximum voltage of 50 kV. All measurements were carried out with a source voltage of 50 kV and source current of 600 μA. Focusing of X-rays was done by a poly-capillary lens, resulting in a final spot 25  μm wide (calibrated for molybdenum Kα radiation).

### (c) Morphometric analysis

Measurements of cupules and ovules were obtained based on the morphological descriptors shown in figure 3*i*. The growth pattern of cupules and ovules was evaluated by plotting the measurements of their length or width in a Cartesian coordinate system and by making regression analyses of each two selected variables. First, the paired ovules in a single fertile unit were compared, and then all ovules, including detached ones, were included in a single pool (measurements in electronic supplementary material, table S4).

The shape variation of ovules was studied by outline analysis. Semi-landmarks on 80 specimens were digitalized using the software TpsDig v. 2.31 [22]. First, an outline was drawn along the curved side of an ovule (integument) from the chalaza to distal apex (as shown in electronic supplementary material, figure S7, dotted lines in different stages that represent the half outline of an ovule), and then a total of 20 semi-landmarks were chosen to represent this outline (coordinate values in electronic supplementary material, table S5). The semi-landmark coordinates were analysed in the software PAST v. 3.08 [23]. The differentiation of semi-landmark coordinates derived from size, position and rotation was removed using a Procrustes fitting transformation. Principal component analysis (PCA) was then performed to summarize major variations between different specimens (shapes).

### (d) Three-dimensional virtual modelling and computational fluid dynamics simulation

Virtual models of cupulate ovules of *Pseudosporogonites*, *Zaijunia* and *Elkinsia* were made based on the average size and morphology of the respective taxa in Cinema 4D R20. It should be noted that the compression fossils of *Zaijunia* provide only two-dimensional information. However, there are more than 100 cupulate ovules in our collection, and owing to their presumed ontogenetic variation and their random preservation, different surfaces of ovules were compressed at different angles and exposed from the rock matrix upon subsequent examination. Therefore, it is possible to reconstruct the three-dimensional appearance of these ovules with some degree of confidence.

However, an additional concern is the orientation of ovules with respect to gravity, particularly owing to the absence of a reconstruction of the entire plant. It was assumed that the apices of ovules were oriented upward or nearly so. This assumption is valid provided that the gross morphology of *Zaijunia* was similar to that of *Elkinsia* (see Discussion), which we consider to be a reasonable inference based on the similarity of their cupulate architectures. It is also consistent with the orientations of many extant gymnosperms assigned to ancient lineages, e.g. cycads and *Ginkgo*. It is also worth noting that reversing the orientation of cupulate ovules would have little or no effect on airflow patterns and that at high ambient airflow speeds, the behaviour of very small particulates (e.g. prepollens) in these patterns would be similar (because of their slow terminal settling velocities).

**Figure 4.**
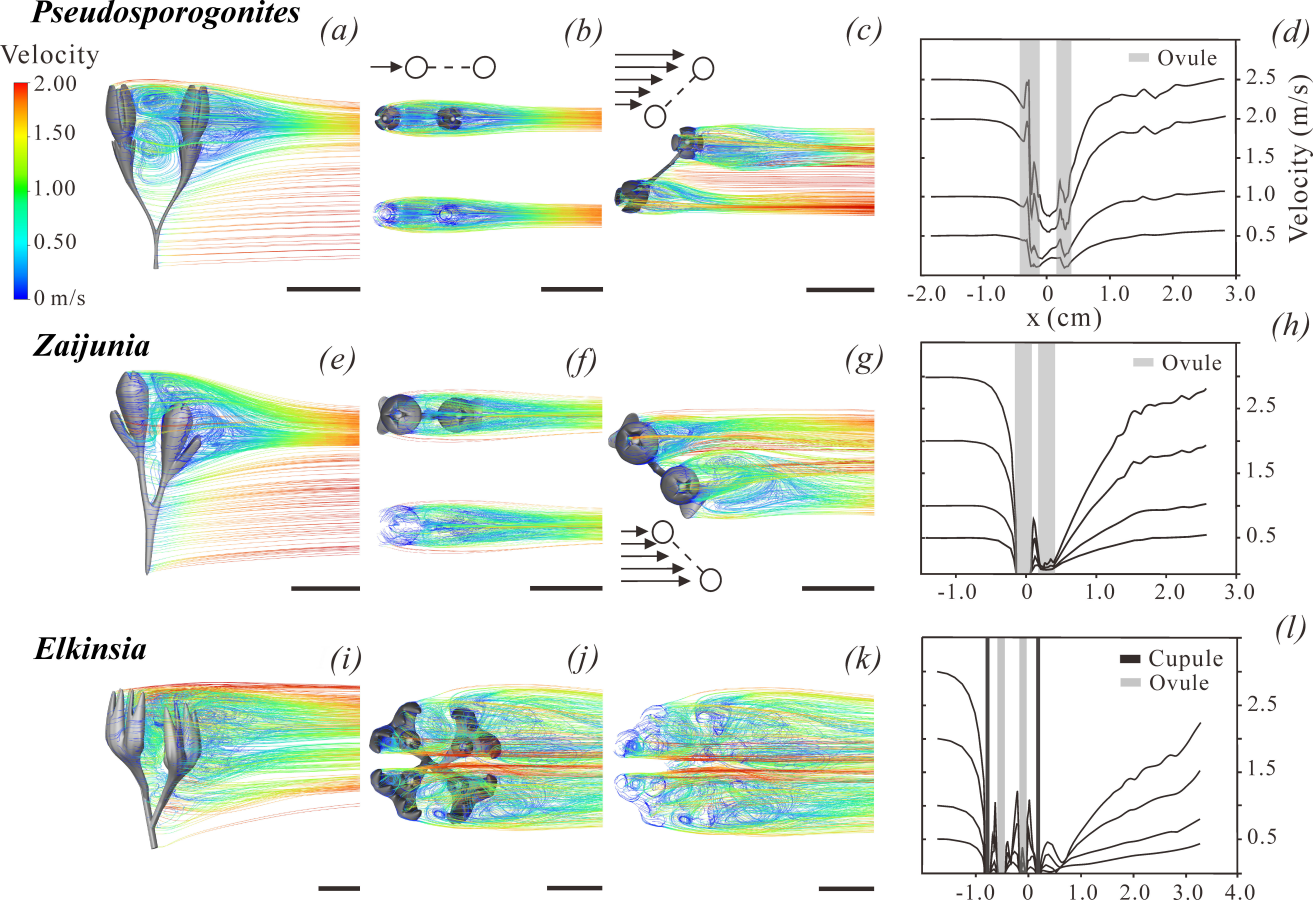
Results of computational fluid dynamics simulations of wind pollination performance of different types of ovules. (*a–d*) *Pseudosporogonites*. (*e–h*) *Zaijunia*. (*i–l*) *Elkinsia*. Pathlines pattern in lateral view (*a,e,i*) and top view (*b,c,f,g,j,k*) of a pair of ovules in *Pseudosporogonites* or of a fertile unit in *Zaijunia* and *Elkinsia*. (*c*,*g*) Pathlines pattern, when wind blows at the ovule model with an angle of 45°. Line drawings indicate the direction of wind (arrows) and ovules (white circles). All models of the ovules are based on their real size. Scale bars, 5 mm (*a–c,e–g,i–k*). All pathlines patterns of flow are coloured according to velocity (colour bar at left side of (*a*)), and the inlet wind velocity is set as 2.0 m s^−1^ (for other inlet velocities, see (*d,h,l*)). Ovule model is deleted in the lower panel in (*b*) for a clearer presentation; same in (*f*) and (*k*). (*d*,*h*,*l*) Estimation of wind velocity along a hypothesized horizontal line through the top of the back ovule in *Pseudosporogonites* and *Zaijunia*, or through the top of one of the back ovules in *Elkinsia*. The horizontal axis (*x*) refers to the distance relative to the models (the positions of ovules and/or cupule are marked by vertical dark lines), and the vertical axis to wind velocity. Four experiments with different inlet wind velocities were performed for each model (data in electronic supplementary material, tables S6–S9). The velocity decreases to 0 when directly encountering the cupules or ovules.

CFD methods were used to demonstrate and quantify the effects of early cupulate ovules on air disturbance. The models were smoothed, repaired and converted in ANSYS SCDM (SpaceClaim) (www.ansys.com). The computational domain consisted of a rectangular prism (electronic supplementary material, figure S10), where the models were centrally fixed and meshed with ANSYS-Meshing. An inlet boundary condition with a normal air inflow velocity and with a turbulence intensity of 0.05 was defined at the upstream end of the domain, and a zero-pressure outlet boundary condition at the downstream end. Simulations of airflow around the three-dimensional models were performed using ANSYS-Fluent 2022 R1 Academic (www.ansys.com) (see electronic supplementary material, videos S1 and S2; figure 4). We validated the CFD parameters by replicating data in the wind tunnel experiments previously performed by Niklas [24]. More information is provided in the electronic supplementary material.

### (e) Phylogenetic analysis

**Figure 5.**
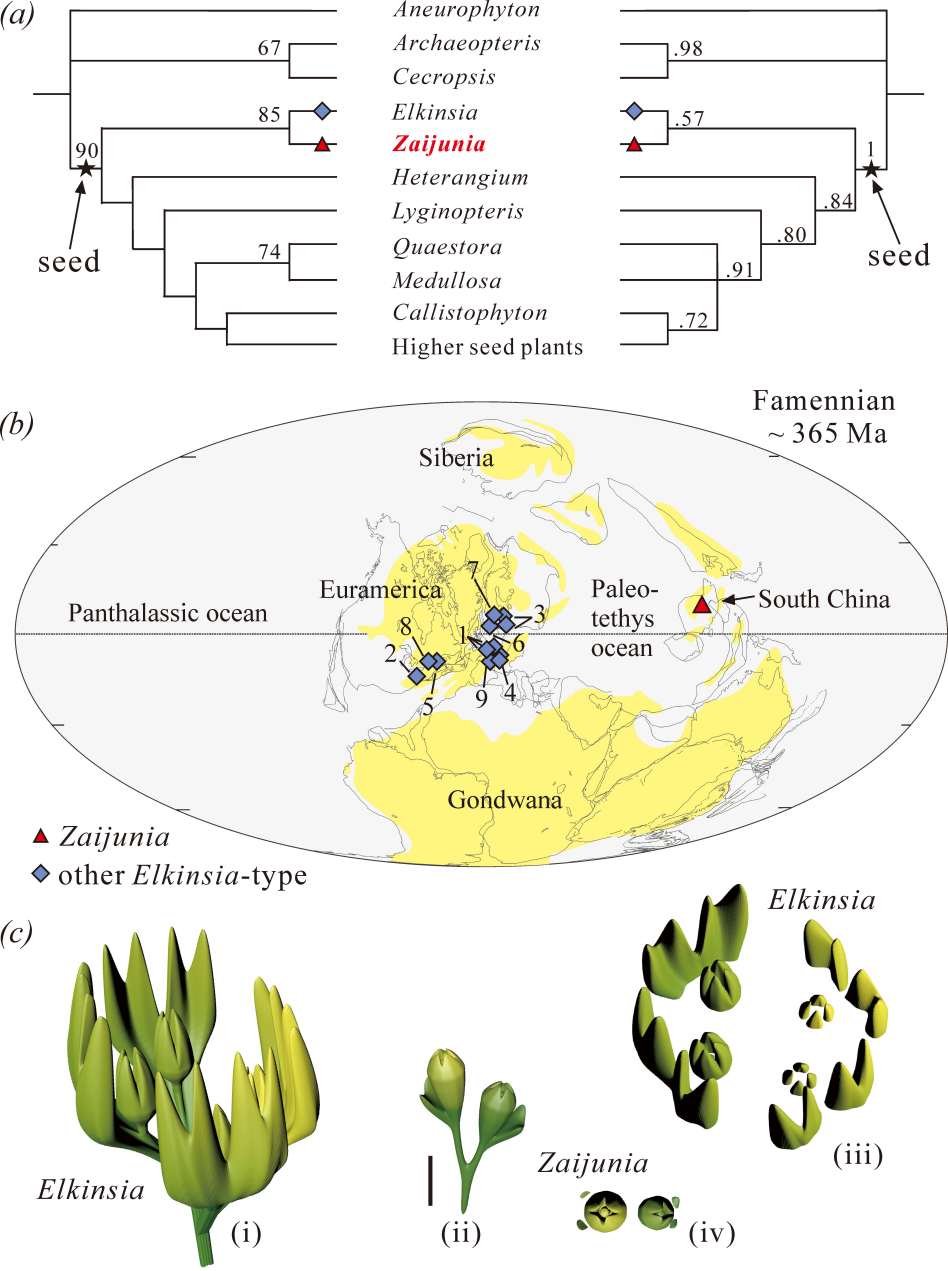
Phylogenetic placement of *Zaijunia* gen. nov. and palaeogeographic distribution of early seed plants with *Elkinsia*-type ovules. (*a*) Simplified cladogram showing the position of *Zaijunia*, as the sister group of *Elkinsia*. The parsimony consensus tree (left, majority-rule consensus method was used) and the Bayesian tree (right) based on the same matrix of 40 plants and 111 characters. Numbers on the branches of the left tree are bootstrap values, and those on the branches of the right tree are posterior probabilities. See electronic supplementary material, figure S11 for the complete trees. (*b*) Distribution of *Elkinsia*-type ovule/seed fossils during the Famennian. The palaeogeographic map based on Scotese [28], with permission. 1, *Moresnetia*; 2, *Elkinsia*; 3, *Xenotheca*; 4, *Condrusia*; 5, *Archaeosperma*; 6, *Kerryia*; 7, *Glamorgania*; 8, *Doudimidia*; and 9, *Thorezia*. Detailed locality information for each genus is provided in electronic supplementary material, table S2. (*c*) Reconstruction of fertile units of *Elkinsia* and *Zaijunia* (i and ii, overall view; iii and iv, top view; scale bar, 5 mm for all).

In order to resolve the phylogenetic position of *Zaijunia*, we used the data matrix of Rothwell & Stockey [25], but made some slight modifications (see electronic supplementary material). The revised data matrix now comprises 111 characters and 40 taxa, which include progymnosperms (*Aneurophyton*, *Archaeopteris* and *Cecropsis*), hydrasperman seed plants (*Zaijunia*, *Elkinsia* and *Lyginopteris*), *Heterangium*, medullosan seed ferns (*Medullosa* and *Quaestora*), callistophytalean seed ferns (*Callistophyton*) and other higher seed plants. The data matrix was analysed with both PAUP 4.0 b 10 using a parsimony method [26] and MrBayes v. 3.2 using a Bayesian inference method [27]. Parts of the resulting trees are shown in figure 5, and the complete trees in electronic supplementary material, figure S11. The modifications of matrix and the procedures are detailed in the electronic supplementary material.

## 3. Results

### (a) Systematic palaeobotany

Division Spermatophyta *sensu* Rothwell & Serbet 1994 [29]

Class Lagenospermopsida *sensu* Cleal 1994 [30]

Order Elkinsiales Rothwell, Scheckler & Gillespie 1989 [6]

Family Elkinsiaceae Rothwell, Scheckler & Gillespie 1989 [6]

Genus *Zaijunia* gen. nov.

**Type species**. *Zaijunia biloba* sp. nov. (figures 1–3*a–c*; electronic supplementary material, figures S3–S7).

**Etymology**. The generic name is derived from ‘Zaijun’, the Chinese style name of Dr Wenjiang Ding, in memory of his early recognition of the Wutong quartz sandstone (now the Wutong Formation) and his numerous and seminal contributions to the establishment of geological disciplines in China.

**Generic diagnosis**. Plant monopodial, with upright stems bearing primary pinnae. Primary rachis bearing multiple secondary pinnae (SPs), or bifurcated to produce two SPs. Tertiary pinnae alternately arranged, bearing alternate pinnules and/or quaternary pinnae. Pinnules planate and highly dissected, one to four times dichotomous. Fertile axes are divided with overtopped, terminal branches bearing fertile units. Each fertile unit consists of a pair of cupulate ovules, with one ovule overtopping the other by means of a longer stalk. Cupules uniovulate, bilobed, with two free tips. Ovules with four integumentary lobes that are acropetally fused approximately four-fifths along their length and then show free tips. Apical portion of nucellus with a hydrasperman-type pollen-receiving mechanism, terminating in a short, tubular salpinx.

***Zaijunia biloba*** sp. nov.

**Holotype designated herein**. PKUB20191 (figure 3*a*).

**Paratypes**. PKUB20121A, B (figure 1*g*,*h*); PKUB20123A, B (figure 1*e*,*f*); PKUB20128A, B (figure 2*a*,*b*); PKUB20142 (electronic supplementary material, figure S5*a*); PKUB20184 (figure 1*a*); and PKUB20190 (figure 3*b*).

**Type locality**. Abandoned quarry near Guangyao village, Changxing county, Zhejiang province, China (electronic supplementary material, figure S1).

**Horizon and age**. The Leigutai Member of Wutong Formation (electronic supplementary material, figure S2); Late Devonian (Famennian).

**Etymology**. The specific epithet refers to the bilobed morphology of the cupules.

**Repository**. The Geological Museum, School of Earth and Space Sciences, Peking University, Beijing, China.

**Specific diagnosis**. Characters as in generic diagnosis. Stems 9.0−12.0 mm wide. Primary rachises 1.5−4.0 mm wide, secondary rachises 0.4−2.5 mm wide, tertiary rachises 0.4−1.0 mm wide and quaternary rachises 0.1−1.0 mm wide. Pinnules 2−10 mm long and 2−13 mm wide. Ultimate fertile branches *ca* 0.5 mm wide, borne on penultimate axes *ca* 2.0 mm wide. Each cupule is oblanceolate in shape, 1.8−7.7 mm long and 0.3−1.2 mm maximum width. Ovules changing in shape during ontogeny, ranging from suborbicular in early stage to obovoid in late stage, 2.0−10.1 mm long and 1.2−3.2 mm in maximum width. Within a fertile unit, longer ovule stalks *ca* 5.0 mm long and shorter stalks *ca* 3.5 mm long. Nucelli 2.8−3.9 mm long and 0.4−1.2 mm wide. Tubular salpinx 0.2−0.7 mm long and 0.3−0.6 mm wide.

### (b) Description of gross morphology

Stems and vegetative and fertile fronds bearing numerous ovules/seeds were collected from a single block of rock. Organic connection among vegetative organs, branching pattern, morphology of pinnae and pinnules and identical striations on all axes indicate that all specimens belong to a single species, although organic connection between vegetative and fertile specimens has not been seen. The well preserved specimen shown in figure 1*a–d* shows the different orders of pinnae, associated with detached ovules. This specimen is interpreted as representing a primary rachis, with up to eight secondary pinnae (SPs) that are attached along its 426 mm length. The proximal two SPs are borne at a short interval (figure 1*a,c*), the third SP extends outward and the fifth to seventh SPs are arranged along one side.

More than 100 cupulate ovules were found, but pollen-bearing organs remain unknown. Although most ovules are detached, some are found in cupulate pairs borne on fertile branches (figure 1*e–h* and figure 2*a–f*; electronic supplementary material, figure S6). In one specimen (figure 1*e,f*), a fertile axis, *ca* 2 mm wide and with fine striations, shows a lateral branch *ca* 0.8 mm wide and 53 mm long bearing a terminal fertile unit, and proximally, two laterals evidenced by their broken bases, which are separated by an interval of *ca* 15 mm. The fertile unit is pendulous, but we consider this to be a preservational accident. Another specimen shows two fertile units subtended by a dichotomous branching system, within which the branches are *ca* 0.5 mm wide; and one fertile unit is obviously overtopping the other (figure 2*a,b*).

A fertile unit shows a basal fork, producing two segments that are unequal in length (figure 1*g, h* and figure 2*a–d*; electronic supplementary material, figure S6). Each segment then divides to produce a cupule and one ovule (figures 1g,h,2j,k). Many specimens show only one cupulate ovule of a pair, with the other ovule having broken away (figure 2*e*,*f*). When completely preserved, the cupule of each ovule further dichotomizes once with a narrow distal angle to form two free tips, thus manifesting a bilobed appearance (figure 2*j,k* and figure 3*a*). In many other specimens, some cupules are laterally compressed and display a swollen structure that closely adheres to one side of its ovule (figure 2*g–i*), and others are separated from their ovules by a narrow gap, with only one cupule tip visible (electronic supplementary material, figure S7*k*,*l*,*n*,*o*). The chalaza of an ovule begins from, or is slightly higher than, the attachment point of its cupule. In lateral view, cupules are oblanceolate in shape and range from 1.8 to 7.7 mm in length and 0.3 to 1.2 mm in maximum width (average = 4.5 mm long and 0.5 mm wide, *n* = 50).

Integuments are smooth and glabrous. Typically, two to four integumentary lobes are visible (figure 2*f–k* and figure 3*a*; electronic supplementary material, figure S7). Nevertheless, inspection of numerous specimens reveals that the norm is four lobes, and the appearance of fewer lobes is due to incomplete preservation. The integumentary lobes are fused throughout most of their length, but free distally for 10−20%. The free integumentary tips are approximately equal in length, 0.5−0.7 mm long, and appear to change little during ontogeny, whereas the whole length of the ovule and its integuments increases significantly (electronic supplementary material, figure S7). The free tips are pointed distally, curving inward, forming a straight or oblique top over the megasporangium (figure 2*g–k* and figure 3*a–c*), but failing to achieve sufficiently close proximity to form a functional micropyle.

A lagenate trace can be found in some ovules (figures 2g, 3b,c), and is interpreted as representing the nucellus. μ-XRF mapping of ovules increases the contrast between nucellus traces and integumentary tissues (figure 3*d–h*; electronic supplementary material, figures S8 and S9). The nucelli are 2.8−3.9 mm long and 0.4−1.2 mm wide (*n* = 7). In one specimen, there is an elliptical impression inside the nucellus trace (figure 3*b*). This impression may represent a macrogametophyte. Future work is required to validate or reject this possibility.

The nucellus terminates in a large, dome-shaped pollen chamber that is surmounted by a salpinx (figure 3*b*,*c*). Proximally, the integument and nucellus are adnate, whereas the nucellus is distally surrounded by integumentary lobes (figure 3*b*). The salpinx is tubular and measures 0.2−0.7 mm long and 0.3−0.6 mm wide (*n* = 4). However, in some specimens, the salpinx shows a slightly expanded top (figure 3*c*). The structure of the pollen chamber is unclear, and no evidence shows the existence of pollination droplets.

Detailed descriptions, illustrations and measurements are provided in electronic supplementary material, figures S3–S9 and table S1.

### (c) Ontogeny of cupules and ovules

The presence of over 100 ovules differing in size makes it possible to propose an ontogenetic sequence. For example, the smallest ovules, which are *ca* 2.0 mm long and *ca* 1.2 mm wide, are suborbicular. As the ovules enlarge, the length increases to as much as 10.1 mm and the width enlarges to 3.2 mm, to produce first an elliptical, and then an elongated obovoid shape (figure 3*l*; electronic supplementary material, figure S7).

The proposed ontogeny is based on the dimensions of three components of the cupulate ovules: (i) the ultimate branch (axis that supports a single cupulate ovule); (ii) the cupule itself; and (iii) the integument. Morphological descriptors of each of these three components are shown in figure 3*i*. Within a fertile unit, the two ovules are comparable in size (figure 3*k*, O1 : O2), and there is a positive correlation between the ovule length and ovule width (figure 3*j* : O versus D), and between ovule length and cupule length (figure 3*j* : O versus C). Although the ultimate branches of a fertile unit with smaller ovules are similar in length, as the ovules enlarge, one of the two ultimate branches increases in length to overtop the other (figure 3*k*, S1 : S2). At maturity, the longer ultimate branches are 3.2−7.6 mm long, and the shorter are 3.0−5.6 mm long. Ultimate branches are *ca* 0.5 mm wide and expand distally to where they attach to the base of the cupulate ovule.

At maturity, one cupulate ovule overtops the other, and there is no concomitant difference in size or morphology. During ontogeny, however, the relative size of the cupule and ovule changes dramatically, with the cupules of small (immature) ovules only slightly shorter than their corresponding ovules, whereas, in larger ovules, the cupules are about one-third of the length of their ovules (electronic supplementary material, figure S7).

### (d) Computational fluid dynamics simulation of aerodynamics of ovules of *Zaijunia* and related taxa

Early seed plants are characterized by a diversity of integument and cupule morphologies [2,3,10–13], which may have affected the capture of airborne (pre)pollen by generating localized regions of turbulent airflow over the apices of ovules [31]. To explore this possibility, we performed CFD experiments to estimate airflow patterns and velocity gradients around three-dimensional digital models of *Zaijunia* cupulate ovules and the ovules of two other seed plants (i.e. *Pseudosporogonites* and *Elkinsia*) (figure 4). The morphology and size of ovules of *Pseudosporogonites* are based on Prestianni *et al*. [11,32], and those of *Elkinsia* are based on Rothwell *et al*. [6] and Serbet & Rothwell [9]. The vertical symmetry axis of the models was fixed perpendicularly to the direction of the wind within the body of influence (b.o.i.) area. Plots of velocity along the apex area of the ovule model and pathlines were used to visualize the results.

Our simulations are consistent with the results of wind tunnel–bubble generator experiments of Niklas [24], but our results demonstrably provide significant advances in spatial and temporal resolution. Nevertheless, it is important to note that all CFD simulations assumed that each (pre)pollen grain is small and light and thus travels within a single air streamline, such that laminar airflow is sufficient to keep (pre)pollen air-borne. Consequently, any localized significant change in airflow, such as turbulence, will discharge suspended (pre)pollen from airflow patterns and thereby permit attachment to a surface.

Overall, CFD simulations indicate low-velocity airflow regions above the apex of the nucellus in all three ovule models of *Zaijunia*, *Pseudosporogonites* and *Elkinsia*. The influence of these regions decreases but becomes more concentrated above the apex as the degree of integumentary lobe fusion increases. Cupules, in particular those with conspicuous lobes, have a pronounced influence on airflow patterns.

In the case of *Zaijunia*, the paired and overtopped arrangement of the two ovules in a single fertile unit has distinctive effects (figure 4*e,f*,*h*), particularly where the taller ovule is upwind of the shorter ovule. In this case, the downwind disturbance in airflow patterns caused by the upwind ovule appears to benefit the shorter downwind ovule by creating a distinctive decrease in air velocity (i.e. as much as *ca* 91% reduction, shown in figure 4*h*), significantly more so than in the case of *Pseudosporogonites* (i.e. as much as *ca* 60% reduction, shown in figure 4*d*). Nevertheless, as the wind direction changes, the paired ovules of *Zaijunia* may become mutually independent (figure 4*g*).

Returning to *Pseudosporogonites*, when there are two ovules in a dichotomous system, the upwind ovule can influence the airflow over the downwind ovule (figure 4*a,b,d*), as in *Zaijunia*. However, as the wind direction changes, the two ovules are almost mutually insensitive to one another (figure 4*c*). The short cupule of *Pseudosporogonites* has little or no demonstrable effect on airflow.

In the more complex fertile unit of *Elkinsia*, which includes a cruciately forked cupule surrounding four ovules, turbulence is predicted to occur on a more significant scale, the large cupule slowing the velocity of air by a magnitude of 80–99% (close to zero near ovule apex; figure 4*i,j*,*l*), regardless of ambient wind direction (figure 4*k*); and thus, compared with *Pseudosporogonites*, a further *ca* 20−39% decrease in air velocity could be produced by the fertile units of *Elkinsia*. In this respect, the aerodynamics generated by the architecture of *Elkinsia* appears to be superior to those of the other two genera examined in this study because it is insensitive to ambient wind direction and yet predicted to be equally efficient at capturing (pre)pollen.

It is important to note that the foregoing simulations did not account for the mechanical properties or behaviour of the long and slender stems subtending ovulate structures (which is a characteristic of many extant wind-pollinated species, e.g. grasses), which may have been capable of harmonic cyclical oscillations, as evidenced by the behaviour of grass peduncles and pedicles [33]. This aspect of modelling calls for a much more elaborate level of computation and requires mechanical parameterization (e.g. the elastic moduli of stems) that is currently unavailable. As already noted, another challenge for our simulations is that the fertile units of *Zaijunia* might be pendulous (although this is less likely when compared with the well preserved specimens of *Elkinsia* [9]). However, even for pendulous fertile units, the disturbance of airflow caused by cupule/integument will surely exist, while the effects of such disturbance on the discharge of (pre)pollen grains need further observation. In all Late Devonian seed plants, documentation of pollination droplets is lacking, even in the permineralized material of *Elkinsia*, and thus the trap mechanism of (pre)pollen grains in these plants remains unclear. However, even if such a trap mechanism should exist, it would show no significant effects on airflow patterns or the behaviour of airborne particulates.

## 4. Discussion

### (a) *Zaijunia* as an early seed plant

Several early seed plants have been described from the Late Devonian (Famennian) deposits around the world [2,10,12,13]. Most are known only for their fertile parts, such as *Archaeosperma* [19], *Moresnetia* [34] and *Pseudosporogonites* [11], although some are more complete, such as the well studied *Elkinsia*, whose vegetative fronds and anatomy have been demonstrated in great detail [6,9], and *Cosmosperma* from South China [12,35]. Based on the configuration of ovules and their cupules, if present, we suggest that the currently known Famennian ovules can be segregated into three main types (electronic supplementary material, table S2). The *Elkinsia*-type is represented by the genera *Elkinsia*, *Moresnetia*, *Archaeosperma* and others and has a widespread distribution particularly in Laurussia (figure 5*b*). In this type, multiple ovules are surrounded by a cupulate system formed by successively cruciate dichotomous divisions, with each ovule laterally subtended in part by the cupule system [10]. The fertile units of *Zaijunia* can be considered the most primitive *Elkinsia*-type (see below). In the *Pseudosporogonites*-type, the cupule is radially symmetrical and located at the base of one or more ovules, or entirely surrounds the ovule(s), as typified by *Pseudosporogonites*, *Dorinnotheca* [36], *Cosmosperma* [12,35], *Latismania* [37] and *Calycosperma* [38]. The third type is the acupulate-type, in which ovules lack cupules or cupule-like structures, e.g. *Guazia* [13] and *Alasemenia* [39].

*Zaijunia* has typical hydrasperman-type ovules (as defined in [2, Fig. 2.2], [3, Fig. 3i], and [9, Figs 52, 54]), with lobed integuments and a complex nucellar apex, similar to the *Elkinsia*- and *Pseudosporogonites*-type ovules. The number of integumentary lobes and the degree of fusion of such lobes varies among different genera; for instance, the integuments of *Moresnetia*, *Elkinsia* and *Archaeosperma* range from completely dissected to nearly completely fused. With respect to integument and nucellus morphology, *Zaijunia* is more similar to *Elkinsia*, because its nucellus is free from the integument along the distal half, with a rounded, dome-shaped pollen chamber surmounted by a short, tubular salpinx.

If we consider the paired ovules of *Zaijunia* as a single developmental and functional unit, each ovule has a lateral lobed cupule, which differs not only from those of the *Pseudosporogonites*-type, but also from the previously known *Elkinsia*-type. *Pseudosporogonites* has isometric paired ovules, each with a small, radially symmetrical cupule (figure 4*a*). *Elkinsia* has loose corymbose tufts of cupulate ovules supported by the overtopped axes of a cruciately forked branch system. The entire cupule system can be divided into two hemispheres, each of which has two cupule quarters (each of which in turn has four free tips and bears one ovule near the base). Thus, a complete cupule system has 16 sterile tips, usually surrounding four ovules (figure 4*i,j*) [6,9]. The morphological comparisons of *Zaijunia* with other taxa are provided in the electronic supplementary material.

Our phylogenetic analyses, based on the data matrix of Rothwell & Stockey [25] with some slight modifications (see electronic supplementary material), produced a parsimony consensus tree and a Bayesian tree, both of which resolve *Zaijunia* and *Elkinsia* as a monophyletic group at the base of the seed plant clade (figure 5*a*; electronic supplementary material, figure S11). Their sister-group relationship is supported by one character, i.e. an ovule enclosed in one or more lateral vascularized structures composed of telomes. In addition, sterile fronds and pinnules of *Zaijunia* and *Elkinsia* are quite similar in morphology (electronic supplementary material, table S3), further enhancing their close relationship. Consequently, we are inclined to assign *Zaijunia* to the family Elkinsiaceae. This clade may also include *Moresnetia*, *Archaeosperma* and *Xenotheca*.

The similarities in the architecture of fertile units of *Zaijunia* and *Elkinsia*, as well as those of their close relatives such as *Moresnetia* [34] and *Archaeosperma* [19], can be interpreted as a reiteration of a modular developmental unit. Each of the paired ovules of *Zaijunia*, with a lobed cupule bearing two free tips, can be considered an equivalent of one hemisphere of the fertile unit of *Elkinsia*, which includes two cupule quarters and two ovules. Also, the paired ovules of *Zaijunia* are quite comparable with the architecture of the cupulate ovules of *Archaeosperma*, in which each of the two hemispheres contains two ovules. Thus, it is plausible that the fertile unit of *Zaijunia* represents one of the simplest architectures of cupulate ovules, and the formation of a more complex derivative *Elkinsia*-type fertile unit might have involved the duplication of *Zaijunia*’s fertile unit (figure 5*c*).

Different scenarios that involve the condensation and fusion of pre-existing systems have also been suggested for other types of cupules [40–42]. Based on anatomically preserved material from the lower Carboniferous, Long [40] proposed that the cupule of *Calathospermum fimbriatum* could have evolved from the condensation and fusion of ‘twin’ cupules or a pair of cupules. The cupule of *Calathospermum* consists of two halves (hemispheres), each half containing multiple ovules and dichotomous cupule lobes. Similarly, cupules containing hydrasperman-type ovules were also hypothesized to have been derived from the fusion of ‘hemi-cupules’ [2,41,42]. Nevertheless, the various terms used in previous literature such as 'twin cupules', 'a pair of cupules', 'cupule halves/hemispheres' and 'hemicupules' indicate the difficulty in recognizing the potential homology.

In summary, the cupulate ovule of early seed plants consists of three parts (or modules), i.e. an ultimate branch (the subtending segment that supports a cupulate ovule), a cupule and an integument. Our review of the literature suggests that the variations of these three modules are more or less independent of one another. For instance, in *Zaijunia,* the cupule is relatively short and simple, whereas in *Elkinsia,* the cupule is a complex branching system with multiple divisions. In at least some groups, the duplication of simple forms, such as *Zaijunia*, might have been an evolutionary pathway leading to more complex forms.

### (b) Adaptation to wind pollination in early seed plants

The morphology of early seed plant reproductive organs, especially the cupules and integuments in different forms, may have been a factor affecting the efficiency of wind pollination [24,31,43,44]. Our computer simulations indicate that cupules and lobed integuments can significantly affect the aerodynamics surrounding these reproductive structures in ways that can direct or bias the deposition of wind-borne (pre)pollen toward the apex of the nucellus, but the patterns are different in different types of cupulate ovules. The architecture of *Elkinsia* is the most sophisticated aerodynamically among the three ‘types’ studied here, and the configurations of *Moresnetia* and *Archaeosperma* are similar to *Elkinsia*, so that their performance in wind pollination may have been similar but also superior to *Zaijunia* and *Pseudosporogonites*. Although other selection pressures may have driven the early rapid radiation of seed plants (e.g. protection and propagule dissemination) [2], we suggest that pollination efficiency was one among them because pollen capture and seed production are inextricably linked. We, like many others, argue that any advantage in seed set, however slight, would have had a cumulative effect over the course of many generations [45,46].

In summary, we speculate that wind pollination efficiency likely played an important role in the diversification of the earliest seed plants in the Late Devonian. This conjecture is based on three features: (i) the general morphology of these taxa is consistent with an anemophilous syndrome (e.g. reproductive organs are borne on long, slender axes lacking vegetative organs such as leaves that can obstruct airflow and impede pollen transport to ovules), (ii) the general (albeit not total) absence of animal (pre)pollen vectors, and (iii) computer simulations showing that cupulate ovule structures can enhance the capture of wind-borne (pre)pollen. Taken in isolation, none of these three features provides a convincing argument. However, when viewed collectively, these features are, at the very least, consistent with the hypothesis proposed here and by others.

## Data Availability

The electronic supplementary material and data supporting this article are available on Figshare: https://doi.org/10.6084/m9.figshare.28091639 [47]. Supplementary material is available online [48].
